# Telemedicine Patient Satisfaction Dimensions Moderated by Patient Demographics

**DOI:** 10.3390/healthcare10061029

**Published:** 2022-06-01

**Authors:** Andrew N. Mason, Matt Brown, Kevin Mason

**Affiliations:** 1 Faculty of Medicine, Juntendo University, 2-1-1 Hongo, Bunkyo, Tokyo 113-8421, Japan; 2 College of Business, Arkansas Tech University, 106 West O Street, Russellville, AR 72801-2222, USA; hbrown11@atu.edu (M.B.); kmason@atu.edu (K.M.)

**Keywords:** telemedicine, patient satisfaction, multi-dimensional measure, gender, income, education

## Abstract

Background: A multi-dimensional telemedicine patient satisfaction measure is utilized to provide managerial insights into where service improvements are needed and factors that impact patient service perceptions. This research explores the influence of patient demographics on telemedicine satisfaction. Four dimensions of telemedicine patient satisfaction (health benefits, patient-centered care, monetary costs, and non-monetary costs) were compared across patient gender, income, and education levels. Methods: A survey of 440 US telemedicine patients on patient satisfaction was measured with Likert scale items to create a multi-dimensional construct using the SERVQUAL model. MANOVA, ANOVA, and linear contrasts were used to examine the impact of patient demographics on telemedicine satisfaction dimensions. Results: The findings revealed that patient demographic characteristics moderated various dimensions of their telemedicine experience satisfaction. Satisfaction with telemedicine health benefits was moderated by patient gender and income levels. Patient-centered care was moderated by patient gender, income, and education levels. Satisfaction with the monetary cost of telemedicine was associated with patient gender and education level. Patient education level influenced their satisfaction with telemedicine non-monetary costs. Discussion: Notable trends include generally higher patient satisfaction for women and those with lower education levels. Patient income showed mixed trends regarding the four dimensions of patient satisfaction. Improvements in patient health literacy along with customized services may improve telemedicine patient care satisfaction and health outcomes. Conclusions: Measuring telemedicine patient satisfaction with a multi-dimensional assessment tool provides insights into how patient demographics influence perceptions of services received. The findings highlighted perceptions of telemedicine patient satisfaction dimensions that differed across patient demographics and provided insights into their overall impact on telemedicine patient satisfaction.

## 1. Introduction

A difficulty facing telemedicine administrators is that measurement of telemedicine patient satisfaction has typically not considered the multiple dimensions that determine overall satisfaction simultaneously. Studies are needed to develop a method to measure telemedicine satisfaction as a multi-dimensional construct. The usefulness of a multi-dimensional measure is that it can provide managerial insights into where service improvements are most needed.

Significant growth in the telemedicine (telehealth) industry continues in the United States (US) [1] and is estimated to exceed a market size of 550 billion dollars by 2027 [2]. Telemedicine services benefit patients and medical providers by minimizing disease exposure to participants, increasing healthcare accessibility, and allowing for more efficient utilization of hospital resources [3,4,5]. In addition, increased reimbursement for telemedicine services and changes in privacy laws are contributing to increased service offerings [6]. Telemedicine services have expanded even more rapidly in recent years because of the COVID-19 pandemic [7].

Patient health is often moderated by patient demographics [8]. For example, coronary heart disease and differential drug metabolism are known to vary based on a patient’s gender [9]. In addition, patient health can be impacted by the patient’s financial affluence and education levels. Financially affluent and higher educated patients tend to exhibit healthier lifestyle habits such as eating better diets and receiving regular medical screenings [10].

In addition, better patient treatment outcomes can be achieved with higher patient satisfaction because satisfied patients are more motivated to adhere to medical advice and regimens [11,12,13,14,15]. Higher patient satisfaction with a medical service is beneficial to the provider as well through higher insurance reimbursements and greater patient loyalty behaviors—both of which lead to increased revenues for the provider [16,17,18]. Personal demographics have been shown to impact service expectations and perceptions, which, in turn, drive satisfaction levels [16]. Thus, patient satisfaction can be moderated by their demographic profile. 

As discussed above, a greater understanding of the patient satisfaction construct can lead to improved patient health and advanced medical services. To understand telemedicine patient satisfaction more fully, factors that moderate this multi-dimensional construct must be examined. The purpose of this study is to examine how various dimensions of telemedicine satisfaction are moderated by patients’ gender, income levels, and education levels.

## 2. Literature Review

Understanding the multi-dimensional nature of telemedicine patient satisfaction is essential for improving patient satisfaction [19,20]. Since patients’ service expectations lead to perceptions of service outcomes, telemedicine patient satisfaction studies often used survey tools where satisfaction was measured with a singular question of whether the patient perceived that the services received met their expectations [21,22,23,24,25,26,27,28,29]. A search of articles indexed with PubMed from 2000 to the present was conducted using the following keywords: “telemedicine patient satisfaction” and “measurement of telemedicine patient satisfaction.” The results revealed that some studies have identified dimensions of the service that impact overall patient satisfaction. For example, telemedicine patient satisfaction has been found to be based upon the effectiveness of service provider communications and/or interactions with the patient [30,31,32]. In other studies, patient satisfaction has been linked to such factors as the healthcare benefits achieved [33], the financial costs, or the time-saving convenience (i.e., non-monetary costs) of the service [27,34]. These previous studies highlight that satisfaction is a multi-dimensional construct [29,35].

SERVQUAL is a methodology commonly used in service-marketing consumer satisfaction research to compute a multi-dimensional satisfaction index by using multiple items to measure various dimensions of a given service [36,37,38]. Recently, Mason presented an adaptation of the SERVQUAL model to examine dimensional aspects of telemedicine patient satisfaction [39]. Mason found that the SERVQUAL model provided reliable and valid patient satisfaction measures and identified four latent dimensions of patient satisfaction. The identified dimensions of patient satisfaction included patient perceptions of the health benefits, patient-centered care, monetary costs, and non-monetary costs associated with services received [39]. More specifically, Mason found that patient satisfaction with telemedicine health benefits was a function of their perceptions of the treatment outcomes. Patient-centered satisfaction was found to be driven by patient perceptions of the provider’s display of empathy and effective interpersonal interactions. Monetary cost satisfaction was found to be based upon patient perceptions of the financial cost savings of telemedicine, and non-monetary cost satisfaction based on a perceived reduction in non-financial costs associated with the telemedicine service such as inconvenience and technology complexities.

Consumer psychology and service marketing literature have found that consumer demographics explain differences in their expectations and perceptions of services [40,41,42,43]. More specifically, gender [44], education level [45,46], and financial affluence [47,48] have all been shown to impact consumer product/service expectations and perceptions. As mentioned earlier, medical research has also shown patient demographics, such as gender and socio-economic characteristics, affect their perceptions of telemedicine services received [8]. With service expectations/perceptions being a driver for satisfaction [16], the following hypotheses were tested:

**Hypothesis** **1** **(H1).** *telemedicine patient satisfaction dimensions are moderated by patient gender*.

**Hypothesis** **2** **(H2).** *telemedicine patient satisfaction dimensions are moderated by patient income levels*.

**Hypothesis** **3** **(H3).** *telemedicine patient satisfaction dimensions are moderated by patient education levels*.

## 3. Materials and Methods

US telemedicine patients served as the study group. The dependent variables were the four dimensions of telemedicine patient satisfaction identified by Mason, which include patient perceptions of the health benefits, patient-centered care, monetary costs, and non-monetary costs associated with the service [39]. The independent variables were patient gender, income, and education level.

### 3.1. Sample

Telemedicine patient survey data was collected by Centiment, an independent survey provider. Centiment solicited input from a patient panel that included 578 recent US telemedicine patients. Recent patients were defined as patients receiving telemedicine services in the past year. Telemedicine patients provided responses to a questionnaire that was created by the authors. The patients were required to provide consent before they participated in the survey. 

### 3.2. Procedures

Prior to collecting data, the authors obtained approval for human subject research from an ethics review board at Arkansas Tech University. Survey data was obtained in April 2021. The data was stored with the lead author and per Institutional Review Board approval regulations is not publicly available. Patient participation was voluntary, and respondents were required to provide participation consent. Respondent anonymity was accomplished by not allowing responding patients the ability to share personally identifiable information. To prevent duplication of respondents, Centiment assigned a custom variable to each respondent entering the survey.

Measurement of the dependent variables (DVs) was conducted with the SERVQUAL instrument used by Mason [39]. Specifically, 7-point Likert scale responses to various statements about the telemedicine service experience. The responses were scaled by anchors where “1” indicated that the respondent strongly disagreed with the statement and “7” indicated strong agreement with the statement. Four items were used to assess patient perceptions of telemedicine service health benefits. Fourteen items were used to assess patient perceptions of patient-centered care. Four items were used to assess patient perceptions of telemedicine monetary costs, and five items were used to measure the telemedicine non-monetary costs. For all DV items, higher scores indicated higher levels of satisfaction. Means were computed from the responses to the individual items associated with a given dependent variable. A total satisfaction outcome was computed as the mean response for all four satisfaction dimensions. The independent variables of patient gender, income and education levels were measured with categorical scales. Gender was measured as male or female; income was measured with six income ranges, and; education level was assessed with four categorical levels (see the Supplementary Material for Questionnaire details).

### 3.3. Statistical Analysis

Multivariate analysis of variance (MANOVA) was used to consider the impact of patient gender, level of income, and education on telemedicine patient satisfaction dimensions. Individual analysis of variance (ANOVA) was used to test for differences within the examined dimensions of patient satisfaction, namely the health benefits, patient-centered care, monetary costs, and non-monetary costs. Additionally, linear contrasts were used to examine the impact of appropriate patient demographics on telemedicine satisfaction dimensions. SAS statistical software was used to conduct all analyses.

## 4. Results

Complete observations from 440 telemedicine patients were obtained, resulting in a seventy-six percent survey response rate. Sixty-three percent of the patients received primary care, twenty-four percent received specialty care, and seven percent had emergency care. In addition, six percent of the patients indicated that they received some other type of telemedicine care.

Patients were evenly divided with fifty percent male and female, respectively. Patient education levels varied with approximately sixty percent holding a least a bachelor’s degree. The median annual household income for participating patients was in the fifty to seventy-five thousand per year range. Overall, the sampled telemedicine patients were consistent with the demographics of telemedicine patients in the US, thus a fairly representative sample [49].

The overall telemedicine patient satisfaction mean observed was 5.1 on the 7.0 scale, where 7 represents the highest possible satisfaction. Thus, the patients’ overall satisfaction observed was consistent with other studies of telemedicine patient satisfaction [50,51]. However, the MANOVA results demonstrated that satisfaction differed across gender, income, and education demographic characteristics. 

The MANOVA analysis results are summarized in Table 1. The four commonly used multivariate statistics, Wilks’ lambda, Pillai’s trace, Hotelling-Lawley trace, and Roy’s greatest root were used to test demographic effects on patient satisfaction. All four multivariate tests showed that gender, level of income, and level of education significantly moderated telemedicine patient satisfaction.

To further explore these differences, separate analysis of variance (ANOVA) results are given for the examined dimensions (see Table 2). Patient gender had highly significant effects on patients’ satisfaction regarding health benefits and patient-centered care (*p* < 0.01 for both) and a marginally significant impact on patients’ satisfaction with telemedicine monetary costs (*p* = 0.06). Likewise, patient income had marginally significant effects on patient satisfaction concerning health benefits (*p* = 0.07) and patient-centered care (*p* = 0.06). Additionally, the effect of patient income on patient satisfaction with telemedicine monetary costs showed some indication of significance (*p* = 0.107). The ANOVA results demonstrated that patient education level significantly impacted satisfaction with monetary and non-monetary costs (*p* < 0.01) and may be related to patient-centered care (*p* = 0.13), however, education level was not found to impact patient health benefits satisfaction.

Linear contrasts were used to test linear trends in patient income and education levels on each of the four dimensions of patient satisfaction (see Table 3). Patient income level had a significant impact on two of the satisfaction dimensions: health benefits and patient-centered care. In addition, patient education level significantly moderated patent satisfaction with telemedicine patient-centered care as well as with monetary and non-monetary costs, respectively. Additionally, patient education level had a marginally significant effect on patient health benefits satisfaction (*p* = 0.097).

The satisfaction means for gender, level of income, and level of education for each of the dimensions are summarized in Table 4, Table 5 and Table 6, respectively. Patient gender significantly moderated telemedicine patient satisfaction; however, the direction of those differences varied by the dimension of patient satisfaction (see Table 4). Overall, females expressed higher telemedicine satisfaction than males. More specifically, female patients had significantly higher satisfaction concerning the health benefits, patient-centered care, and monetary costs dimensions of patient satisfaction. Female patients also had higher satisfaction with the non-monetary costs of telemedicine, although the satisfaction difference was not significant.

Likewise, dimensions of patient satisfaction significantly varied by patient income levels (see Table 5). Patient satisfaction with telemedicine health benefits and patient-centered care were more favorable as the patient’s level of income increased. However, patient income did not significantly moderate satisfaction with the monetary costs and non-monetary costs of telemedicine.

The level of education obtained by patients significantly moderated their satisfaction for select dimensions of telemedicine patient satisfaction. As shown in Table 6, patient education level had a significant impact on patient satisfaction with patient-centered care, monetary costs, and non-monetary costs. Specifically, while patients with the lowest education level were an anomaly, an examination of the overall linear trends revealed that higher satisfaction with telemedicine patient-centered care, monetary costs, and non-monetary costs were observed for patients with lower educational levels. Significant differences in telemedicine patient satisfaction for telemedicine health benefits were not found to be related to the patient’s level of education.

## 5. Discussion

The MANOVA findings demonstrate that patient telemedicine satisfaction dimensions are moderated by patient gender (H1), income level (H2), and education level (H3). Thus, the results supported all three hypotheses. In addition, patient demographic effects on patient satisfaction dimensions varied depending on the demographic examined.

Female and male patients both had favorable overall telemedicine services satisfaction levels. However, female patients were significantly more satisfied with the health benefits and patient-centered care associated with telemedicine services. Female patients had their lowest satisfaction with the monetary costs followed by the non-monetary costs of telemedicine. Therefore, specialty providers in women’s care (e.g., Obstetrics and Gynecology), may be able to improve satisfaction more with a focus on keeping monetary costs low followed by improving non-monetary costs such as patient convenience and comfort. 

The findings also show areas for satisfaction improvements are greater for male versus female patients across all dimensions. For example, the lowest satisfaction dimension observed was for male patients’ perceived satisfaction with monetary cost, which may indicate that male patients have greater price sensitivity to telemedicine. In addition, satisfaction with patient-centered care was significantly lower for male patients versus females. While medical science training is vitally important for providers, medical staff must also be trained in the “soft skills” associated with effective interpersonal communications and attitudes toward patient care. The findings imply that the “soft skills” currently being employed by providers during telemedicine encounters are not as well received by their male patients and may need improvement. The key to improving patient-centered care is to focus on relationships between providers and patients. Providers should focus on what matters to the patient as much as what is wrong with the patient. Patient-centered care requires that providers communicate healthcare information in layman’s terms and get their male patients engaged in the decision-making while ensuring that the patient has control over their care. Findings in this study indicate that providers are doing a better job of building patient-centered care relationships with female patients, yet relationship building is equally important for male patients [52].

Patients with higher incomes also appear to have greater satisfaction with the health benefits and patient-centered care associated with their telemedicine service. On the other hand, with regards to patient education, the more highly educated patients had lower satisfaction with telemedicine patient-centered care and costs, both monetary and non-monetary. Less educated patients appeared to be more satisfied with these three dimensions than the more educated patients. This may reflect that as education level increases so do critical reasoning and expectations. Thus, the less educated may be more easily satisfied with the interpersonal communication “soft skills” of the medical staff. More educated patients may be more critical and may have higher standards (expectations) and so may represent a challenge regarding their personal care expectations. This may also explain the higher satisfaction with monetary and non-monetary costs that lower educated patients display, as patients that are more satisfied with the patient-centered care received may feel that the price they pay (both monetary and non-monetary) is commensurate with the value they receive from the provider interaction.

For all patient demographics examined, unrealistic expectations or confusion regarding the patient-provider interaction, resulting from low patient health literacy, may account for some of the differences in patient satisfaction observed. Poor patient health education is a recognized factor in medical services in that approximately thirty-three percent of all US patients lack adequate understanding of health information and how to properly follow medical advice [53]. Telemedicine is naturally more impersonal and difficult to communicate information than via a face-to-face meeting. Those with higher health literacy are likely better equipped to grasp important medical information being conveyed by their providers and, as a result, may be more satisfied with perceived health benefits and patient-centered care of providers. On the contrary, to those with lower health literacy, health information conveyed by the provider may be more difficult to grasp, and thus they experience frustration, leading to lower levels of satisfaction with perceived health benefits and patient-centered care. This in turn may have a downstream effect on patients’ perceived satisfaction with monetary and nonmonetary costs.

The concept of patient health education will likely grow in importance as healthcare moves from the traditional “doctor knows best” approach to a patient-centered care focus [52]. As such, healthcare providers are encouraged to tailor communications to meet the patients’ level of understanding. For example, to improve health benefits and patient-centered care, medical staff should avoid using medical jargon. Rather, healthcare providers should provide information and instructions in smaller concrete points and use visual aids, when possible. Then, to ensure patient comprehension, ask the patient to explain their understanding of the information. Medical providers must engage their patients on a personal basis to motivate them to better healthcare understanding and motivate them to treatment adherence. 

To enhance patient-centered care, medical service providers should seek ways to get patients more engaged and invested in their health. One way to improve patient engagement with medical services may be with the use of apps that run on smart devices (i.e., smartphones, tablets, etc.). Apps have been used to improve patient health acumen and engage them to monitor disease treatments and, thus, help them to follow treatment regimens accurately. For example, there is an FDA-approved EKG app that detects atrial fibrillation from home or work that provides the patient and physicians with a comprehensive assessment of cardiac health.

Future research is needed to improve the understanding of patient satisfaction as well as to generate a consensus around a generally accepted patient satisfaction measurement method. The SERVQUAL model, used in this study, offers a useful multi-dimensional method to measure telemedicine patient satisfaction. The SERVQUAL model allows for investigation into multiple aspects of telemedicine experiences, which can lead to targeted improvements in services. As such, the authors recommend the use of the SERVQUAL model in future patient satisfaction research. 

To expand telemedicine patient satisfaction knowledge, studies are also needed using the SERVQUAL model to examine patient satisfaction across other potentially moderating factors such as culture or type of healthcare service (e.g., emergency care, primary care, specialty care, etc.). In addition, research is needed to compare gender price sensitivity differences across cultures to compare whether gender monetary satisfaction perceptions differ across countries where healthcare is socialized versus where it is not.

## 6. Conclusions

Telemedicine patient satisfaction is a salient construct that impacts patient medical treatment adherence, thus patient health. In addition, satisfied telemedicine patients may be inclined to use future telemedicine services or recommend them to others, thus leading to sustainable revenues for providers. The study highlights the benefits of measuring telemedicine patient satisfaction as a multi-dimensional construct to identify where targeted improvements are needed. The findings also highlight the need for telemedicine providers to customize services according to their patients’ demographic profiles.

Findings also underscore the importance of measuring telemedicine patient satisfaction as a multi-dimensional construct. For example, patient gender was found to moderate satisfaction with health benefits, patient-centered care, and monetary costs. Patient income was found to moderate satisfaction with health benefits and patient-centered care. Finally, patient education was found to moderate patient-centered care, monetary costs, and non-monetary costs. Variations in satisfaction across the observed demographics provide a nuanced understanding of the drivers of patient satisfaction. 

## 7. Limitations

In addition to its observational nature, the study is limited by an inability to include all possible factors of interest. As a result, there is a potential for unobserved variables to impact the generalization of these results. For example, the patients sampled are from multiple telemedicine providers, and the difference in providers could not be considered in this study. Additionally, the specific reasons respondents were seeking healthcare were not considered. Despite the limitations, the moderating impact of patient demographic information on patient satisfaction indicated in previous studies was shown through the innovative use of the SERVQUAL model. The impact of additional factors and their possible interaction with these moderating factors are potential avenues for future research.

## Figures and Tables

**Table 1:** MANOVA Results.

Moderating Factor	Wilks’ Lambda (*p* > F)	Pillai’s Trace (*p* > F)	Hotelling-Lawley Trace (*p* > F)	Roy’s Greatest Root (*p* > F)
Gender	0.0069	0.0069	0.0069	0.0069
Income Level	0.0381	0.0406	0.0364	0.0002
Education Level	<0.0001	<0.0001	<0.0001	<0.0001

**Table 2:** ANOVA Results for Dimensions of Patient Satisfaction.

Moderating Factor.	ANOVA: Health Benefits (*p* > F)	ANOVA: Patient-Centered Care (*p* > F)	ANOVA: Monetary Costs (*p* > F)	ANOVA: Nonmonetary Costs (*p* > F)
Gender	0.0016	0.0026	0.0607	0.1502
Income Level	0.0700	0.0605	0.1071	0.9650
Education Level	0.3359	0.1348	<0.0001	<0.0001

**Table 3:** Linear Contrast Results for Dimensions of Patient Satisfaction.

Moderating Factor	Linear Contrast: Health Benefits (*p* > F)	Linear Contrast: Patient-Centered Care (*p* > F)	Linear Contrast: Monetary Costs (*p* > F)	Linear Contrast: Nonmonetary Costs (*p* > F)
Income Level	0.0198	0.0156	0.9611	0.6052
Education Level	0.0977	0.0327	0.0001	0.0004

**Table 4:** Means for Dimensions of Patient Satisfaction by Patient Gender.

Gender	Mean: Health Benefits ^a^	Mean: Patient-Centered Care ^a^	Mean: Monetary Costs ^b^	Mean: Nonmonetary Costs	Grand Average	Sample Size
Female	5.51	5.97	4.61	4.91	5.25	220
Male	5.26	5.78	4.17	4.59	4.95	220

**^a^** Significant Difference (*p* < 0.05). **^b^** Marginally Significant Difference (*p* = 0.0607).

**Table 5:** Means for Dimensions of Patient Satisfaction by Patient Income Level.

Level of Income	Mean: Health Benefits ^a^	Mean: Patient-Centered Care ^a^	Mean: Monetary Costs	Mean: Nonmonetary Costs	Grand Average	Sample Size
>$100,000	5.56	6.02	4.07	4.59	5.06	145
$75,000 to $100,000	5.45	5.90	4.24	4.71	5.08	91
$50,000 to $74,999	5.25	5.81	4.82	4.86	5.19	66
$35,000 to $49,000	5.17	5.72	4.85	4.79	5.13	48
$20,000 to $34,999	5.32	5.73	4.61	4.90	5.14	57
<$20,000	5.19	5.75	4.30	4.96	5.05	33

**^a^** As income increased, satisfaction significantly increased (*p* < 0.05).

**Table 6:** Means for Dimensions of Patient Satisfaction by Patient Education Level.

Level of Education	Means for Health Benefits	Means for Patient-Centered Care ^a^	Means for Monetary Costs ^a^	Means for Nonmonetary Costs ^a^	Grand Average	Sample Size
Doctorate	4.98	5.47	3.68	4.16	4.57	24
Master’s	5.48	5.92	3.71	4.17	4.82	113
Bachelor’s	5.46	5.98	4.77	5.03	5.31	169
High School or Less	5.30	5.78	4.60	4.98	5.17	134

**^a^** As education level increased, satisfaction decreased (*p* < 0.05).

## Data Availability

The data that support the findings of this study are available on request from the corresponding author. The data are not publicly available due to privacy or ethical restrictions.

## Supplementary Materials

The following supporting information can be downloaded at: https://www.mdpi.com/article/10.3390/healthcare10061029/s1, Survey Questionnaire.
