# Supplementary material for: Telemedicine Patient Satisfaction Dimensions Moderated by Patient Demographics
Source: Healthcare (Basel). 2022 Jun 1;10(6):1029. doi: 10.3390/healthcare10061029 (PMC9223157; doi:10.3390/healthcare10061029)
Supplement: Supplementary file 1 [file healthcare-10-01029-s001.zip › healthcare-1735085-supplementary.pdf]

Supplemental Material: Survey Questionnaire

Introduction and Respondent Consent

You are invited to participate in this study which is about telemedicine healthcare service satisfaction. The survey takes between 5 to 10 minutes to complete. You may participate if you are 18 years of age or older. Your participation in this study is completely voluntary. There are no foreseeable risks associated with this project. However, if you feel uncomfortable answering any questions, you can withdraw from the survey and all previous data you provided will be deleted.

Your survey responses will be strictly confidential and data from this research will be reported only in the aggregate. Your information will be coded and will remain confidential. If you have questions at any time about the survey or the procedures, you may contact Dr. Andrew Mason by email at m.andrew.at@juntendo.ac.jp. If you have questions regarding your rights as a research participant or you have concerns or general questions about the research, contact the Arkansas Tech University Institutional Review Board Programs at irb@atu.edu.

Please click on the statement that best describes your consent to participate in this survey.

____ I wish to participate in this survey.

____ I do not wish to participate in this survey. If you check this response, you will close the survey link.

Please click on the statement that best describes your age.

____ I am at least 18 years old. Clicking this response takes you to the survey.

____ I am under 18 years old. Clicking this response ends the survey.

For this next section, please consider your most recent experience with telemedicine service and rate your level of agreement to the following statements on a seven-point scale where 1=Strongly Disagree; 2=Disagree; 3=Somewhat Disagree; 4=Neutral; 5=Somewhat Agree; 6=Agree; and 7=Strongly Agree.

Dependent Variables

Health Benefits – mean computed from four statements.

1. Telemedicine provides greater access to healthcare specialists. (i.e., *specialist access*).
2. Telemedicine provides greater overall healthcare access. (i.e., *overall access*).
3. Telemedicine provides safety from exposure to diseases. (i.e., *disease exposure*).
4. Telemedicine provides excellent healthcare. (i.e., *healthcare*).

Patient-Centered Care - mean computed from fourteen statements.

1. The telemedicine healthcare providers were very knowledgeable. (i.e., *provider knowledge*).
2. The telemedicine healthcare providers were very courteous. (i.e., *provider courtesy*).
3. The telemedicine healthcare providers were very credible. (i.e., *provider credibility*).
4. The telemedicine healthcare providers were very trustworthy. (i.e., *provider trustworthy*).
5. The telemedicine healthcare providers accurately communicated important aspects of the service in a way I could understand. (i.e., *information*).
6. The telemedicine healthcare providers were responsive to my needs. (i.e., *responsive*).
7. I am confident that telemedicine healthcare providers will protect my privacy. (i.e., *privacy*).
8. The telemedicine healthcare providers created an atmosphere for positive communications. (i.e., *communications*).
9. The telemedicine healthcare providers create an atmosphere for positive relationships. (i.e., *positive relationships*).
10. The telemedicine healthcare providers were very caring. (i.e., *caring providers*).
11. The telemedicine healthcare providers were very attentive to my needs. (i.e., *attentive providers*).
12. The telemedicine healthcare providers were very interested in my health. (i.e., *patient health*).
13. The telemedicine healthcare providers were very focused on my best interests. (i.e., *patient interest*).
14. The telemedicine healthcare providers listened carefully to me. (i.e., *provider listened*).

Monetary Costs - mean computed from four statements.

1. Telemedicine costs less than traditional healthcare service. (i.e., *lower costs*).

2. Telemedicine reduces complications with the healthcare insurance processes. (i.e., *insurance complexities*).
3. Telemedicine increases healthcare insurance coverage for patients. (i.e., *insurance coverage*).
4. Telemedicine reduces expenses. (i.e., *expense*).

<u>Non-Monetary Costs</u> – mean computed from five statements.

1. Telemedicine makes appointment scheduling more convenient. (i.e., *scheduling*).
2. Telemedicine decreases wait time for services. (i.e., *wait time*).
3. Telemedicine equipment used by patients is not complex. (i.e., *service complexities*).
4. Telemedicine equipment requirements are not problematic. (i.e., *equipment problems*).
5. Telemedicine technology is non-intimidating. (i.e., *intimidating technology*).

<u>Overall Telemedicine Satisfaction -</u> mean computed by averaging Health Benefits, Patient -Centered Care, Monetary Costs, and Non-Monetary Costs. (i.e., *total satisfaction*).

<u>Independent Variable Items</u>

1. Gender (i.e., *gender*) - measured as:
   - -Male
   - -Female
   - -Prefer to not answer

2.. Household annual household income (i.e., *income*) - measures as:
   - -Less than $20,000
   - -$20,000 to $34,999
   - -$35,000 to $49,999
   - -$50,000 to $74,999
   - -$75,000 to $100,000
   - -Over $100,000

3. Highest level of education (i.e., *education*) - measured as:
   - -High school degree or less
   - -Bachelor's degree
   - -Master's degree
   - -Doctorate degree
